# To select relevant features for longitudinal gene expression data by extending a pathway analysis method

**DOI:** 10.12688/f1000research.15357.1

**Published:** 2018-07-31

**Authors:** Suyan Tian, Chi Wang, Howard H. Chang

**Affiliations:** 1 Division of Clinical Research, The First Hospital of Jilin University, Changchun, Jilin, 130021, China; 2 Department of Biostatistics, The University of Kentuchy, Lexington, KY, 40536, USA; 3 Department of Biostatistics and Bioinformatics, Emory University, Atlanta, GA, 30322, USA

**Keywords:** Core subset; feature selection; gene set analysis; longitudinal microarray data; significance analysis of microarray (SAM)

## Abstract

The emerging field of pathway-based feature selection that incorporates biological information conveyed by gene sets/pathways to guide the selection of relevant genes has become increasingly popular and widespread. In this study, we adapt a gene set analysis method – the significance analysis of microarray gene set reduction (SAMGSR) algorithm to carry out feature selection for longitudinal microarray data, and propose a pathway-based feature selection algorithm – the two-level SAMGSR method. By using simulated data and a real-world application, we demonstrate that a gene’s expression profiles over time can be considered as a gene set. Thus a suitable gene set analysis method can be utilized or modified to execute the selection of relevant genes for longitudinal omics data. We believe this work paves the way for more research to bridge feature selection and gene set analysis with the development of novel pathway-based feature selection algorithms.

## Introduction

The emerging field of pathway-based feature selection that incorporates biological information conveyed by gene sets/pathways to guide the selection of relevant genes ^1, 2^ has become increasingly popular and widespread. Here, a gene set or a pathway refers to a collection of genes that function together to influence and even regulate a specific biological process. In this study, the phrases “gene set” and “pathway” are used interchangeably.

Since biological systems are dynamic, researchers are extremely interested in investigating gene expression patterns over a time course, in an effort to capture dynamical changes that are biologically meaningful and have casual implications. With the fast evolution of microarray technology and RNA-Seq technology, longitudinal experiments that collect gene expression profiles over a series of time points have become affordable and increasingly common in the fields of biomedicine and life science.

The analytical strategy typically employed for such longitudinal data is to stratify the whole dataset into separate subsets according to time points and then analyze the resulting subsets separately. This approach fails to consider the correlations among measures of a specific subject at different time points. Additionally, it overlooks those genes with trivial changes at individual points but non-marginal accumulated effects when taken together. Therefore, this approach is usually inefficient and lacks statistical power ^3– 5^.

On the other hand, some statistical methods that can analyze longitudinal gene expression data directly have been proposed. Among them, many have adopted a filter method to carry out the selection of relevant features for longitudinal gene expression profiles by screening genes one by one. For example, the GEE-based screening procedure by ^5^ fits a GEE model ^6^ to each gene and then excludes those non-significant genes (i.e., a gene with the corresponding p-value/q-value is larger than a pre-determined cutoff). By filtering genes one by one, this GEE-based screening is highly likely to include many redundant genes and thus to inflate the false positive rate. The redundant genes are irrelevant but suggested to be associated with the phenotype of interest by a feature selection method, mainly due to their correlations with the true relevant genes. Another example is the EDGE method proposed by Storey *et al.* ^3^. The EDGE method is designed to identify differentially expressed genes over time between different phenotypes. This method utilized spline-based models to construct expression value-versus-time curves for individual genes and then screened genes one by one according to their significance levels. Again, this method has the same drawback as the GEE-based screening does, namely, the inclusion of many redundant genes.

To the best of our knowledge, there is no pathway-based feature selection algorithm for longitudinal gene expression data. Given the fact the pathway-based feature selection methods have been demonstrated to be superior to the conventional feature selection methods, there is an urgent need to develop pathway-based algorithms, in order to tackle longitudinal data.

Here, we propose one extension to a pathway analysis method – significance analysis of microarray gene set reduction (SAMGSR) ^7^ to conduct feature selection for longitudinal microarray data. In this modification, we extend SAMGSR by applying its reduction step twice. At the first reduction step, the core gene subsets corresponding to the selected gene sets are identified. Then, the essential time points of the selected genes are obtained subsequently. This extension is referred to as the two-level SAMGSR algorithm hereafter.

## Methods

A previous version of this article is available as a pre-print on arXiv at Cornell University Library: https://arxiv.org/ftp/arxiv/papers/1511/1511.08272.pdf. In that version, the two-level SAMGSR algorithm and another extension we have made to the SAMGSR algorithm for longitudinal feature selection were included. After the preprint submission, we had made substantial modifications. Also we realized that the updated manuscript with two extensions together was easy to confuse the readers. Therefore, we decided to describe two extensions in separate manuscripts.

### Experimental data

Data for the injury experiment were downloaded from the Gene Expression Omnibus repository. The accession number is GSE36809. This experiment was hybridized on Affymetrix HGU133 plus2 chips.

In this study, only patients with uncomplicated recoveries and patients with complicated recoveries were considered. According to Xia *et al*. ^8^, uncomplicated recovery represents a recovery within 5 days while complicated recovery includes a recovery after 14 days, no recovery by 28 days, or death. If the recovery duration is longer than 14 days, the patient experienced complicated recovery for sure. Therefore, the possible time points for an uncomplicated recovery include days 1/2, 1, 4, 7 and 14, whereas those for a complicated recovery are days 1/2, 1, 4, 7,14, 21, and 28. Furthermore, we restricted our focus to the patients that had the full compliment of measurements (i.e., complicated patients with 7 measures and uncomplicated patients with 5 measures). The 25 uncomplicated patients and 18 complicated patients who met this request were put together and used as the training set. Since there were no measures for uncomplicated patients after 14 days, the data for patients with complications were truncated at 14 days.

The rest of patients including 50 uncomplicated patients and 23 complicated patients were used as a test set to validate the proposed method. In the test set, the time points considered were days 1/2, 1, 4, and 7. Of note, the characteristics of patients in the training set and the test set may be different since the test set includes patients who had been discharged early from the hospitals.

### Pre-processing procedures

Since different pre-processing procedures may impact the data analysis, we decided to download the summary expression values of the experimental data directly from the GEO database.

### Pathway information

The gene sets were downloaded from the Molecular Signatures Database (MSigDB) ( http://software.broadinstitute.org/gsea/msigdb). In this study, we considered the C2 category in this knowledgebase, which includes gene sets from curated pathways databases such as KEGG ^9^ and those manually curated from the literature on gene expression.

### Statistical methods

Here, we present a brief introduction to the SAMGSR algorithm ^7^, and then discuss our extension for the purpose of feature selection for longitudinal gene expression data in detail.

***SAMGSR.*** The SAMGSR algorithm is an extension of the SAMGS method ^10^ and provides additional reduction of significant gene sets into respective core subsets. According to Dinu *et al*. ^7^, the SAMGSR method may result in an approximately 90% of reduction in the size of selected genes, in an effort to improve predictive performance and allow biological patterns to become more obvious.

The SAMGSR method consists of two major steps: the SAMGS process to identify important gene sets and the reduction step to refine those significant gene sets into their respective core subsets. In the SAMGS step ^10^, an SAMGS statistic, the L _2_ norm of the SAM statistics ^11^ for all genes within that gene set, is calculated. A p-value of the SAMGS statistic is computed using a permutation test by permuting phenotype labels of samples. Based on the p-value, the statistical significance of a gene set is determined. Second, focusing on those significant gene sets, the reduction step orders genes within a significant gene set *j* based on the magnitude of its SAM statistic and then gradually partitions the entire gene set into two subsets: the reduced subset R _k_ which includes the first k genes with largest SAM statistics, and the residual subset ${\bar{R}}_{k}$ being the complement of R _k_ for k=1,…, |S-1|. Here S is the size of gene set *j*. Let c _k_ be the SAMGS p-value of the residual subset ${\bar{R}}_{k}$. The optimal size of reduced set R _k_ is the smallest k such that c _k_ is larger than a pre-specified cut-off for the first time.

**Figure 1.**
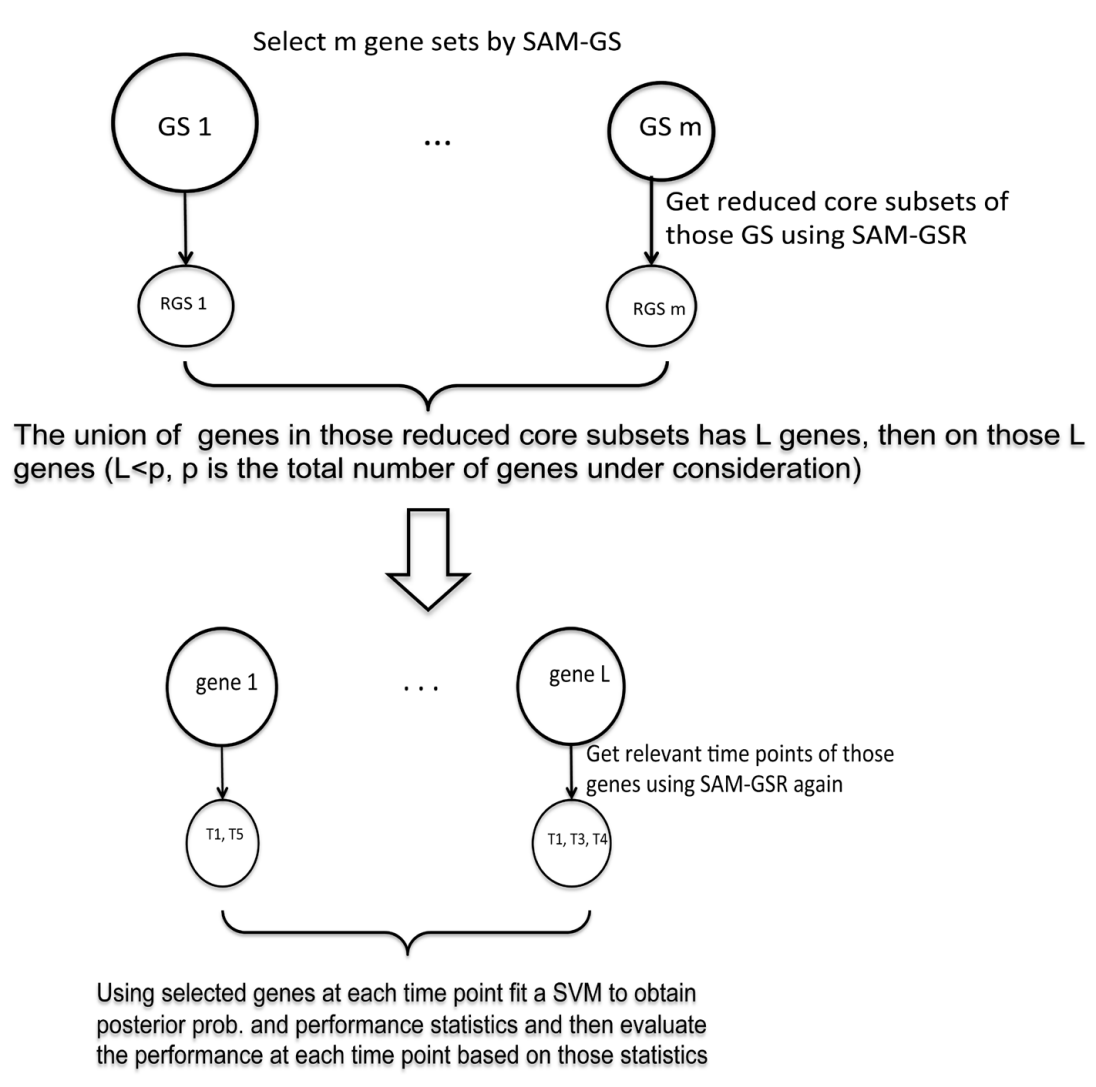
Flowchart illustrates the two-level SAMGSR algorithm.

***SAMGSR extension for longitudinal feature selection.*** In our modification to the SAMGSR method for the purpose of longitudinal feature selection, a gene set has two different meanings. First, it refers to a set of genes in a curated biological pathway. Second, it refers to a gene’s expression profiles across time. Correspondingly, the reduction step of SAMGSR is applied in an ordered manner at two levels: a lower level on gene sets and an upper level on the time points. First, the reduction procedure of SAMGSR is applied to identify the reduced core subsets within the selected/reduced gene sets obtained by the SAMGS step. Then upon the union of genes involved in those core subsets, the reduction procedure is applied again to determine exact time points where the expression values of those genes differ significantly between phenotypes. In the two-level SAMGSR algorithm, the SAMGS statistic is modified to as,

$$SAMGS_{GS_{k}}=\sum_{i=1}^{\left|GS_{k}\right|}\sum_{j=1}^{t_{i}}d_{ij}^{2},d_{ij}=({\bar{x}}_{d}(ij)-{\bar{x}}_{c}(ij))/(s(ij)+s_{0j})$$

where d _ij_ is the SAM statistic ^11^ of gene i (i=1,…, |GS _k_|) at time point j (j=1,…, t _i_) and |GS _k_| is the size of gene set GS _k_ (k=1,…,K, and K is the total number of gene sets). ${\bar{x}}_{d}(ij)$ and ${\bar{x}}_{c}(ij)$ are the sample averages of gene i at time point j for the diseased group and the control group, respectively. Parameter s(ij) is a pooled standard deviation that estimated by pooling all samples together, while s _0j_ is a small positive constant used to offset the small variability in microarray expression measurements. Of note, both s(ij) and s _0j_ are time-point specific because the variability of expression measurements differs at different time points. In the reduction step, SAMGS is calculated sequentially first to a series of subsets (for the size of 1,…, |GS _k_|-1) of a significant gene set, aiming to identify a core subset that makes an essential contribution to the statistical significance of this gene set. Then the algorithm moves to the level of time points, with the objective of determining which combination of time points contributes substantially to the importance of the specific gene. At this level, each gene’s expression profiles over time were viewed as a gene set. Our rational is that a gene’s expression values for the same individual over time are highly correlated, mimicking a gene set. Figure 1 elucidates the two-level SAMGSR algorithm.

In a separate study (unpublished study; Suyan Tian, Chi Wang, Howard H. Chang), we proposed another extension to the SAMGSR method for the purpose of longitudinal feature selection, which is referred to as the longitudinal SAMGSR method. The longitudinal SAMGSR method first applies the SAMGS step to select the relevant genes and then determines exact time point(s) that the expression values for a gene differ between two phenotypes. A potential disadvantage of this SAMGSR extension is it does not incorporate valuable biological information contained in pathways, which provides knowledge on how genes function in unison to impact on biology processes.

In both SAMGSR extensions, c _k_ is regarded as a tuning parameter. Using the sequence of 0.05, 0.1, …, 0.5, the optimal value of c _k_ corresponds to the one associated with the minimum 5-fold cross-validation (CV) error. In a 5-fold CV, a dataset was randomly divided into 5 roughly equal-sized folds, and 4 of these folds were used to train a classifier and the misclassification error rate was calculated upon the held-out fold. This step was repeated for each of the 5 folds as the held-out fold, and the error rates were averaged. Given the fact the SAMGSR extensions cannot estimate the coefficients of selected genes, support vector machine (SVM) models were fitted to estimate those coefficients. Then the posterior probability for a sample can be calculated for each time point.

### Performance metrics

In this study, we use four metrics - Belief Confusion Metric (BCM), Area Under the Precision-Recall Curve (AUPR), Generalized Brier Score (GBS), and the misclassified error – to evaluate the performance of a classifier. Our previous study ^1^ provided detailed descriptions on those metrics. In summary, all these metrics have a range in between 0 and 1. For the first two, the closer to 1 the better a classifier is. In contrast, a value of 0 is optimal for the last two metrics. Given the SAMGSR extensions tend to identify those genes that are insignificant at isolated time points but significant jointly over time, an evaluation on individual time points using these statistical metrics might be unfair for the SAMGSR extensions, we also averaged the resulting posterior probabilities at each time point and then calculate the performance metrics using those averages.

### Statistical language and packages

Statistical analysis was conducted in the R language version 3.1.2 ( www.r-project.org). The Venn-diagram plot was made with the aid of an online bioinformatics tool. R codes of the two-level SAMGSR algorithm are given in the Supplementary File 1.

## Results and Discussion

### Real world application

Traumatic injury with subsequent infection was a common cause of death in ancient times. Even today massive injury such as combat wounds remains life threatening ^12, 13^. A large clinical study that examined the genome-wide expression patterns of blood leukocytes in the immediate post-injury period was carried out several years ago ^8^. A primary objective of that study was to explore if different patterns of gene expression existed for the two extremes of clinical recovery: the uncomplicated recovery and the complicated recovery. We used the longitudinal gene expression data collected specifically for this objective to evaluate our proposed method.

First, the comparison between the two SAMGSR extensions and the SAMGSR separately at each time point was made. The two-level SAMGSR extension incorporates both the interaction information among genes inside a pathway and the correlations among the expression values of one specific gene over time. In contrast, the longitudinal SAMGSR extension only accounts for the correlations among the expression values of one specific gene over time, while the application of SAMGSR at individual time points only considers the interactions among genes inside a pathway. This comparison allows us to identify which factor - the interactions among genes inside pathways or the correlations among the same gene over time have significant impact on the performance of resulting signatures. The results were given in Table 1, from which we found the two-level SAMGSR method performs the best at the second and third time points while the longitudinal SAMGSR method does so at the first and fourth time points. Overall, the implementation of SAMGSR at separate time points has the worst performance.

**Table 1.** Performance of the SAMGSR algorithm and our SAMGSR extensions for longitudinal feature selection, evaluating on individual time points.

	5-fold CV					Test set			
	Day 1/2	Day 1	Day 4	Day 7	Day 14	Day 1/2	Day 1	Day 4	Day 7
A. Using two-level SAMGSR (94-gene signature, cutoff for c _k_ = 0.2 on the whole training set) ^1^									
# of genes	40.6	31.6	33.2	38.6	45	63	55	49	63
GBS	0.304	0.266	0.306	0.274	0.278	0.298	0.272	0.240	0.288
BCM	0.514	0.565	0.536	0.556	0.526	0.491	0.534	0.560	0.495
AUPR	0.533	0.690	0.610	0.617	0.575	0.494	0.551	0.594	0.527
B. Using simple SAMGSR (97-gene signature, cutoff for c _k_ = 0.2 on the whole training set) ^1^									
# of genes	51	32.6	35	36.2	39.2	69	53	45	77
GBS	0.279	0.210	0.279	0.323	0.262	0.262	0.309	0.307	0.257
BCM	0.501	0.598	0.501	0.498	0.559	0.499	0.513	0.498	0.534
AUPR	0.551	0.739	0.514	0.522	0.609	0.503	0.521	0.514	0.572
C. Using SAMGSR at each time point (the size of signature >1000, cutoff for c _k_ = 0.1 on the training set) ^1^									
# of genes	230.2	23	59	74.6	453.6	360	30	61	42
GBS	0.257	0.231	0.327	0.305	0.272	0.264	0.295	0.266	0.296
BCM	0.506	0.551	0.478	0.497	0.520	0.491	0.486	0.515	0.492
AUPR	0.535	0.655	0.482	0.518	0.584	0.490	0.482	0.529	0.512

Note: ^1^ the posterior probabilities were calculated using an SVM classifier. Here, the cutoff for q-value in SAM-GS part is set at 0.05. # of genes represents the average number of genes over 5-fold cross-validated data selected by an algorithm at each time point for the five columns on the training set.

As we mentioned in the previous section, a separate evaluation on an algorithm at single time points might be unfair for both SAMGSR extensions, and an integrated evaluation that takes all time points into account is necessary. Therefore, we did such analysis. When taken all time points together, the superiority of the longitudinal SAMGSR method over the two-level SAMGSR method and the original SAMGSR method at separate time points has been established. The respective performance statistics are provided in Table 2.

**Table 2.** Performance of the SAMGSR algorithm and our SAMGSR extensions for longitudinal feature selection, when all time points considered together.

Method	# of genes	5-fold CV				Test set			
		Error	GBS	BCM	AUPR	Error	GBS	BCM	AUPR
Two-level SAMGSR ^1^	94	0.419	0.258	0.507	0.541	0.356	0.239	0.525	0.566
L-SAMGSR ^1^	97	0.442	0.268	0.515	0.576	0.356	0.230	0.535	0.622
SAMGSR separately ^1^	>400	0.419	0.246	0.510	0.559	0.428	0.243	0.511	0.553

Note: ^1^ the posterior probabilities were calculated using an SVM classifier. Here, the cutoff for q-value in SAM-GS part is set at 0.05. # of genes represents the number of the union of individual genes selected at each time point. L-SAMGSR: the longitudinal SAMGSR method.

In summary, incorporation of the pathway information inside gene sets, the clusters of genes that might be potentially co-expressed/co-regulated together, did not result in the two-level SAMGSR having substantially superior performance. One possible explanation relates to the information quality of the pathway database itself. The canonically curated databases on pathways/gene sets are biased toward the well-studied diseases such as cancers, and with substantial less works investigating traumatic injury using gene expression profiles, the pathway knowledge contained inside those curated pathways may be uninformative for this specific disease.

**Figure 2.**
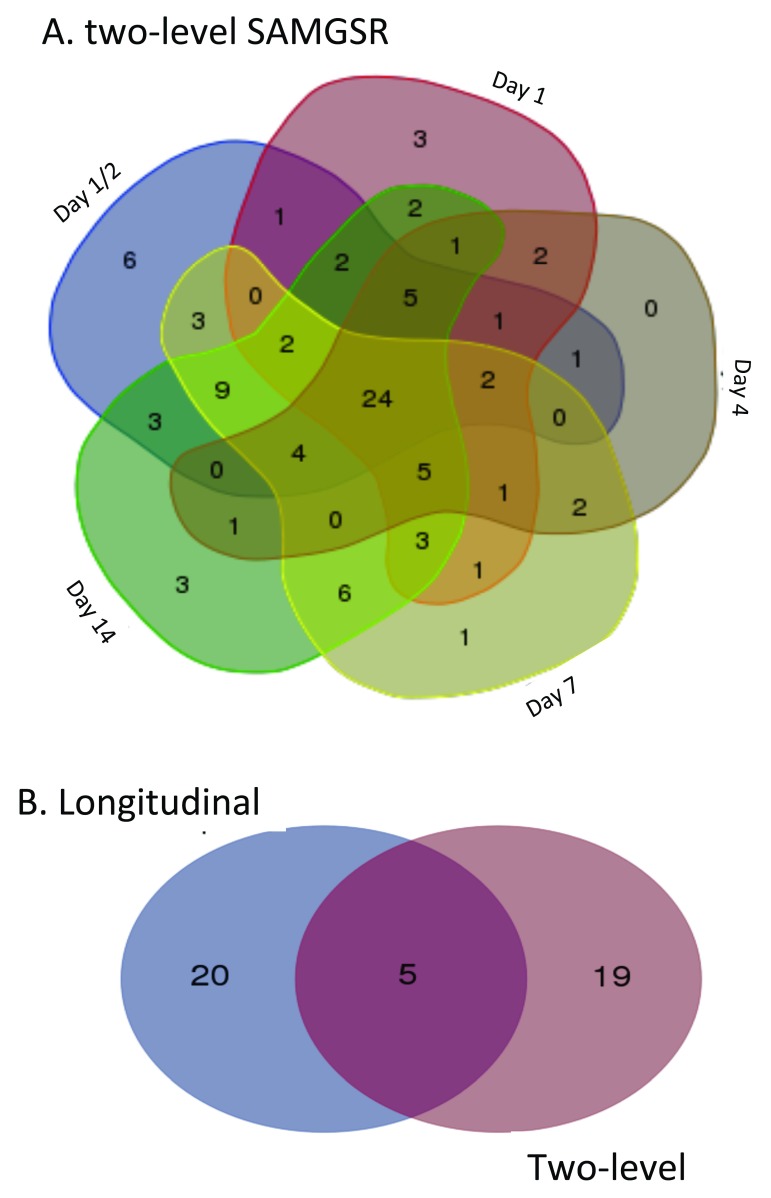
Selected genes by the two-level SAMGSR algorithm in the traumatic injury application. ( **A**) Venn-diagram illustrates the overlap of selected genes by the two-level SAMGSR method at different time points. ( **B**) Venn-diagram illustrates the overlap of concordantly differentially expressed genes across all time points by the two-level SAMGSR algorithm and the longitudinal SAMGSR algorithm.

In Figure 2, Venn-diagrams illustrate how the selected genes by the two-level SAMGSR method at different time points overlap. We observed that these two SAMGSR extensions perform comparably in term of overall model parsimony. Namely, the two-level SAMGSR algorithm identifies 94 unique genes while the longitudinal SAMGSR algorithm identifies 97 unique genes. Moreover, we observed that in the 94-gene signature identified by two-level SAMGSR, there is a substantial proportion of overlaps at all time points (24/94; 25.53%), while the number of genes being significant only at one specific time point is one half of this number. Again, this highlights the ability of our SAMGSR extensions (both the longitudinal SAMGSR method and the two-level SAMGSR method) to identify genes that present mild but concordant change across time points between two different phenotypes.

**Figure 3.**
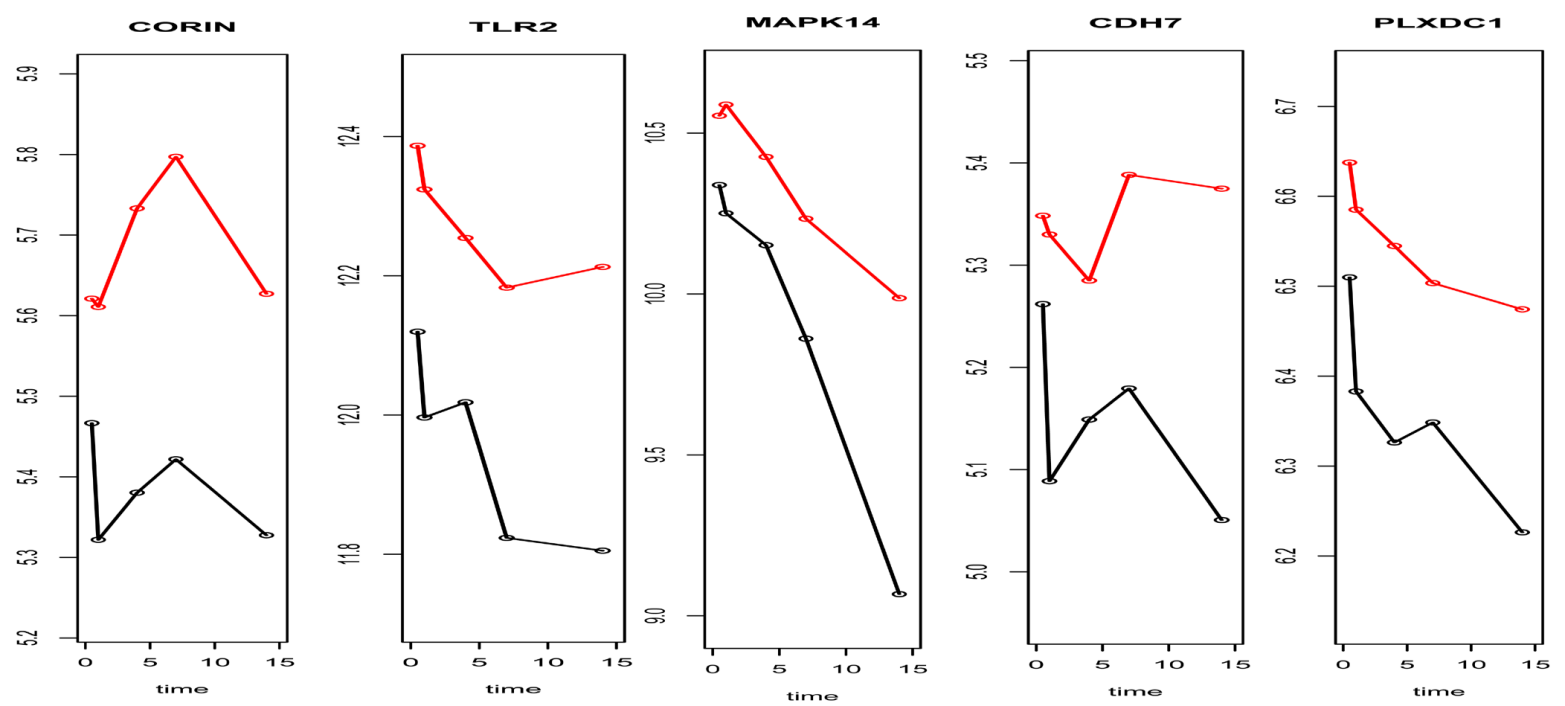
Characteristics of 5 common significant expressed genes across all time points by both two-level SAMGSR method and the longitudinal SAMGSR in the traumatic injury application. Subgroup sample means versus time plot for the 5 common genes that were identified as to be significant at all 5- time points between uncomplicated and complicated patients. Red line represents the complicated group while black line represents the uncomplicated group.

Upon the overlapped 5 genes by both SAMGSR extensions, a plot ( Figure 3) was made to compare the expression patterns over time between the complicated injury and the uncomplicated injury. It is observed that the pattern of change across time points for complicated patients versus uncomplicated ones is not quite unique or simple. Thus the results of our analysis provide no evidence on either the paradigm that complicated outcomes are associated with second hits or multiple inflammatory events which thus subsequently cause a secondary genomic response ^14, 15^, or its counterpart stating complication results from simultaneous and rapid induction of innate and suppression of adaptive immunity genes ^8^. Further investigation is in demand.

### Simulated data

In order to investigate the properties of both SAMGSR extensions, we used observed expression values from the injury application to design two sets of simulations as in our previous study. Briefly, we chose two causal genes – F13A1 and GSTM1 – and then randomly selected 998 genes from the data serving as noise in the first simulation setting. Denote the expression value of gene *i* (F13A1 or GSTM1) at time *j* (j=1,…, 5) as X _i.j_, the logit function of a complicated injury versus an uncomplicated injury is as following,

            log *it _clu_* = 0.18 *X _F_*_13_ *_A_*_1.1_ + 0.57 *X _F_*_13_ *_A_*_1.2_ + 0.29 *X _F_*_13_ *_A_*_1.3_ + 0.41 *X _F_*_13_ *_A_*_1.4_ + 1.02 *X _GSTM_*_1.3_

Here, we considered one gene whose significance arises from its moderate joint contribution over time and the other whose association with the outcome is large at one specific time point. The aim of this simulation was to illustrate the inferred advantage possessed by the two SAMGSR extensions, namely, both of them incorporate the accumulated effect of genes over time, recognizing genes with mild or moderate change at each time point but with a coordinated change over time.

In the second simulation, we chose two genes – COX4I2 and RP9 as the relevant genes. The logit function was,

                    log *it _clu_* = 0.56 *X _COX_*_4_ *_I_*_2.1_ − 0.91 *X* *_RP_*_9.5_

For both simulation settings, 50 replicates were created. The results for these two simulations are given in Table 3 and Table 4, respectively. Unexpectedly, the longitudinal SAMGSR showed no inferiority to the two-level SAMGSR in both correctly selecting relevant genes and achieving a better model parsimony. Regarding model parsimony, the inferiority of two-level SAMGSR may stem from the pathway level; a relevant gene would be involved in many gene sets. Consequently, the number of highly correlated genes with the relevant ones might increase and since these genes are included in the final model, the parsimony of two-level SAMGSR naturally suffers. Regarding correct selection of causal genes, since two simulations are based on the injury data in which the biological knowledge might add no extra value to feature selection, as shown in the real-world application, it is unsurprising to have both algorithms correctly identify the causal genes.

**Table 3.** The results of simulation 1.

Method		Time 1	Time 2	Time 3	Time 4	Time 5
L-SAMGSR (Ave. # 32.06)	# of genes	19.84	19.14	13.68	9.30	11.00
	F13A1	72 %	100 %	100 %	92 %	68 %
	GSTM1	0 %	0 %	62 %	22 %	0 %
Two-level SAMGSR (Ave. # 61.74)	# of genes	38.88	32.66	21.44	18.96	20.50
	F13A1	64 %	92 %	90 %	84 %	52 %
	GSTM1	2 %	62 %	94 %	80 %	36 %

Note: # of genes represents the average number of genes selected by either the longitudinal SAMGSR algorithm or the two-level SAMGSR algorithm at each time point over 50 replicates. Ave # represents the average number of unique genes across 5 time points. The percentages of the causal genes being correctly selected at each time point over these 50 replicates are presented in the corresponding cells.

**Table 4.** The results of simulation 2.

Method		Time 1	Time 2	Time 3	Time 4	Time 5
L-SAMGSR (Ave. # 291.98)	# of genes	182.38	56.18	35.44	30.94	123.84
	COX4I2	96 %	0 %	0 %	0 %	4 %
	RP9	10 %	4 %	4 %	6 %	96 %
Two-level SAMGSR (Ave. # 327.56)	# of genes	209.44	73.40	48.04	49.38	138.66
	COX4I2	100 %	0 %	0 %	0 %	0 %
	RP9	4 %	0 %	0 %	0 %	92 %

Note: # of genes represents the average number of genes selected by either the longitudinal SAMGSR algorithm or the two-level SAMGSR algorithm at each time point over 50 replicates. Ave # represents the average number of unique genes across 5 time points. The percentages of the causal genes being correctly selected at each time point over these 50 replicates are presented in the corresponding cells.

Although in the second simulation the number of relevant time points was less than that in the first one, the number of selected genes by both algorithms was dramatically larger in the second simulation. This might be because the relevant genes in the second simulation were highly correlated with other genes compared to the first simulation. The highly correlated structure between relevant features and irrelevant ones produced a large number of redundant features that both SAMGSR extensions, especially the two-level SAMGSR, cannot exclude. To our best knowledge, however, many feature selection algorithms, especially those based on filtering, may suffer from this drawback. As illustrated in our previous work ^16, 17^, an additional filtering using a relevant algorithm such as bagging ^18^ may provide a solution to alleviate or eliminate this problem.

## Conclusions

Both of the SAMGSR extensions incorporated the correlated structure of an expression’s profiles over time in the framework of gene sets, and were more likely to identify genes with coordinated and aggregated effects over time, while their effect size at individual time points may be insignificant. The naïve strategy of implementing feature selection separately at individual time points would overlook these genes. The employed process explains why the overlaps among the selected genes by both extensions over time were very large.

The curated pathways in major databases such as KEGG ^9^ and GO ^19^ tend to be enriched in the most prevalently studied diseases, e.g., cancers. Moreover, the pathways are far from completeness even for these diseases ^20^. These facts potentially introduce biases and unfairness to an algorithm that utilizes pathway information to guide feature selection, e.g., the two-level SAMGSR method. One solution is to consider a statistical method to construct data-driven gene sets e.g., 21. Future work to construct such gene sets for longitudinal microarray data is needed, particularly to determine whether gene sets are stable or dynamic over time. Based on these facts, we suggest the longitudinal SAMGSR algorithm should be considered first, especially for an entry-level data analyst. If the diseases under investigation are cancers or the lab has its own customized pathways for the diseases, the two-level SAMGSR algorithm is recommended because the biological information contained in those pathways could provide more values on selecting relevant genes.

In this article, we adapted the SAMGSR method for feature selection of longitudinal gene expression profiles. To the best of our knowledge, this study is one of the few efforts to explore how to execute feature selection for longitudinal gene expression data, with additional consideration on pathway knowledge. Given that the two-level SAMGSR extension only performs comparable at individual time points and is even outperformed by the longitudinal SAMGSR method when considerer all time points together, our try here is not fruitful. Nevertheless, we believe this work paves the way for more research to incorporate pathway information to guide feature selection, with the development of novel algorithms to tackle longitudinal gene expression data.

## Data availability

The microarray data were downloaded from the Gene Expression omnibus (GEO) repository, accession number GSE36809. The R codes of the two-level SAMGSR algorithm were given in Supplementary File 1.

## Supplementary material

**Supplementary File 1:** The R-codes for the two-level SAMGSR method.
