# Supplementary material for: To select relevant features for longitudinal gene expression data by extending a pathway analysis method
Source: F1000Res. 2018 Jul 31;7:1166. [Version 1] doi: 10.12688/f1000research.15357.1 (PMC6124382; doi:10.12688/f1000research.15357.1)
Supplement: Supplementary file 1 [file f1000research-7-16734-s0000.tgz › 379fe3e0-e77d-4739-ac1e-bbc58b98000e.docx]

Supplementary Materials

# R codes for the Two-level SAMGSR algorithm

# the outer loop for biological pathways in MSigDB)

hiearSAMGS <- function(GS,DATA, tp,

cl, nbPermutations=1000,

silent=F, lambda=seq(0, 0.9, 0.05), pi0.method = "smoother"){

GS <- GS.format.dataframe.to.list(GS)

if(!silent) print("GS dataframe-to-list : done.")

genes <- unique(intersect(dimnames(DATA)[[1]],unlist(GS)))

genes <- genes[!is.na(genes)]

nb.Samples <- ncol(DATA)/tp

nb.GeneSets <-length(GS) #nb of gene sets

GeneSets.sizes <- sapply(GS,length) #size of each gene set

C1.size <- table(cl)[1]

samT.ok<-NULL

s0.time<-NULL

DATA <- DATA[genes,]

for (j in 1:tp){

# finding constant s0 for SAM-like test

DATA.temp<-DATA[, which(((1:ncol(DATA))%%(-tp)+tp)==j)]

tmp<- sam.TlikeStat(DATA.temp,cl=cl)

s0 <- tmp$s0

if(!silent) print("s0 estimation : done.")

samT.ok <-c(samT.ok, as.data.frame(tmp$TlikeStat)[genes,]) # SAM T-like statistic for each gene

s0.time<-c(s0.time, s0)

}

samT.ok.temp<-matrix(unlist(samT.ok), tp, nrow(DATA), byrow = TRUE)

sam.sumsquareT.ok.i <- apply( samT.ok.temp,2, function(z) sum(z^2)) # SAMGS statistics for each gene over time

names(sam.sumsquareT.ok.i)<-as.character(rownames(DATA))

sam.sumsquareT.ok.o<- sapply(GS, function(z) sum(sam.sumsquareT.ok.i[z]))

C2.size<-table(cl)[2]

nb.Samples<-length(cl)

# stats obtained on 'permuted' data

permut.C1 <- matrix(NA,nbPermutations,C1.size)

sam.sumsquareT.permut.o<- matrix(NA,nbPermutations,nb.GeneSets)

diperm.ts<-matrix(NA,(dim(DATA)[1]*tp), (nbPermutations+1))

diperm.ts[,1]<-unlist(samT.ok)

diperm<-matrix(NA,(dim(DATA)[1]), (nbPermutations+1))

diperm[,1]<-sam.sumsquareT.ok.i

for(i in 1:nbPermutations) {

C1.permut <- permut.C1[i,] <- sample(nb.Samples,C1.size)

C2.permut <- (1:nb.Samples)[-C1.permut]

samT.permut<-NULL

for (j in 1:tp){

DATA.temp<-DATA[, which(((1:ncol(DATA))%%(-tp)+tp)==j)]

samT.permut <- c(samT.permut, as.data.frame(sam.TlikeStat(DATA.temp,C1=C1.permut,C2=C2.permut,s0=s0.time[j])$TlikeStat))

}

diperm.ts[,(i+1)] <-unlist(samT.permut)

# SAMGS statistics for each gene set - for current permutation

junk<-apply(matrix(unlist(samT.permut), tp, nrow(DATA), byrow = TRUE),2, function(z) sum(z^2))

diperm[,(i+1)]<-junk

names(junk)<- as.character(rownames(DATA))

sam.sumsquareT.permut.o[i, ]<-sapply(GS, function(z)sum(junk[z]))

if(!silent & i%%50 == 0)print(paste(i," permutations done."))

}

GeneSets.pval <- apply(t(sam.sumsquareT.permut.o) >= sam.sumsquareT.ok.o ,1,sum)/nbPermutations

names(GeneSets.pval)<-names(GS)

qobj <- NULL

try(qobj <- qvalue(GeneSets.pval, lambda=lambda,pi0.method=pi0.method))

GeneSets.qval <- rep(NA,nb.GeneSets)

# PI0 <- NA

if(!is.null(attr(qobj,"class"))){

GeneSets.qval <- qobj$qvalues

}

res <- as.data.frame(cbind("GS size"= GeneSets.sizes,

"GS p-value (0 <=> < 1/nb permutations)" = GeneSets.pval,

"GS q-value"= GeneSets.qval))

res <- cbind(res,"GS name"= names(GS))[c(4,1:3)]

L <- list("GS stats"=res, "diperm"=diperm, "diperm.ts"= diperm.ts)

L

}

#Other related functions for SAMGSR (downloaded from Dr. Yasui’s homepage)

rowMeansVars <- function(d,margin=1){

if(margin==2) d <- t(d)

m <- rowMeans(d,na.rm=T)

dif <- d - m

ssd <- rowSums(dif^2,na.rm=T)

list("means"=m,

"sumsquaredif" =ssd,

"vars" =ssd/(ncol(d)-1),

"centered rows"=dif )

}

#SAM statistics

sam.TlikeStat <- function(DATA, cl=NULL, C1=NULL,C2=NULL,

s0=NULL, s0.param=list(nb.Groups=100,mad.Const=.64),

alternative=c("two.sided", "greater", "less")[1], conf.level =0.95 ){

if(!is.null(cl)){

cl <- as.factor(as.character(cl))

C1 <- which(as.numeric(cl)==1)

C2 <- which(as.numeric(cl)==2)

}

if(is.null(C1) | is.null(C2))stop("Error -sam.TlikeStat : classes 1 and 2 are undefined.")

nb.Genes<- nrow(DATA)

nb.Samples<- ncol(DATA)

C1.size<- length(C1)

C2.size<- length(C2)

stat.C1<- rowMeansVars(DATA[,C1])

stat.C2<- rowMeansVars(DATA[,C2])

diffmean.C1C2<- stat.C1$means - stat.C2$means

pooledSqrtVar.C1C2<-sqrt((1/C1.size+1/C2.size)*(stat.C1$sumsquaredif+stat.C2$sumsquaredif)/(nb.Samples-2))

if(is.null(s0)){

nb.Groups <- s0.param$nb.Groups

mad.Const <- s0.param$mad.Const

tmp <- as.data.frame(cbind(pooledSqrtVar.C1C2,diffmean.C1C2))

tmp <- tmp[order(tmp[,1]),]

group.Size<- as.integer(nb.Genes/nb.Groups)

percentiles<- seq(0,1,.05)

nb.Percentiles <- length(percentiles)

s0.quantiles<- quantile(pooledSqrtVar.C1C2,percentiles)

tt <- matrix(NA,nb.Groups,nb.Percentiles)

coeffvar <- as.data.frame(cbind(s0.quantiles,rep(NA,nb.Percentiles)))

for(j in 1:nb.Percentiles){

x<-matrix(tmp[1:(group.Size*nb.Groups),1]/(tmp[1:(group.Size*nb.Groups),2]+ s0.quantiles[j]),group.Size,nb.Groups)

tt[,j]=apply(x,2,mad,constant=mad.Const,"na.rm" =TRUE)

coeffvar[j,2] <- sd.na(tt[,j])/mean.na(tt[,j])

}

s0 <- min(s0.quantiles[coeffvar[,2]==min(coeffvar[,2])])

}

tstat <- diffmean.C1C2/(pooledSqrtVar.C1C2+s0)

df <- nb.Samples-3 # <- s0 is a supplemental parameter to estimate

if (alternative == "less") {

pval <- pt(tstat, df)

cint <- cbind(rep(-Inf,nb.Genes), tstat + qt(conf.level, df) )

}else if (alternative == "greater") {

pval <- pt(tstat, df, lower = FALSE)

cint <- cbind(tstat - qt(conf.level, df), rep(Inf,nb.Genes))

}else {

pval <- 2 * pt(-abs(tstat), df)

alpha <- 1 - conf.level

cint <- qt(1 - alpha/2, df)

cint <- cbind(tstat -cint,tstat + cint)

}

list(s0= s0,

diffmean = diffmean.C1C2,

pooledSqrtVar = pooledSqrtVar.C1C2,

TlikeStat = tstat,

"p.values (using Student law)"=pval,

gm.C1 = stat.C1$means,

gm.C2 = stat.C2$means,

confidence.intervals=cint)

}
