# Supplementary material for: To select relevant features for longitudinal gene expression data by extending a pathway analysis method
Source: F1000Res. 2018 Jul 31;7:1166. [Version 1] doi: 10.12688/f1000research.15357.1 (PMC6124382; doi:10.12688/f1000research.15357.1)
Supplement: Peer review history

# Peer review: Referee response for version 1

**DOI:** 10.5256/f1000research.16734.r37148

**Published:** 2018-09-12

**Authors:** Irina Dinu

**Affiliations:** 1 school of public health, University of Alberta, Edmonton, AB, Canada

1. Considering the intense method developments in time-course microarray data analysis, the authors should include a more rigorous literature review in background section supporting their claim about the existence of no pathway-based feature selection method.
2.   As the proposed method is selecting both significant genes and significant time-points, the authors should explain “feature selection” in introduction in order to clarify the study objectives for the reader.
3.  In method description, the authors should describe how they calculated p-value and explain the corresponding permutation procedure in each of the steps.
4. This method is able to detect the genes with different “levels” of expression over time and it fails to detect differential temporal patterns of gene expressions. The figure 3 also shows that the significant genes are different in expression levels and similar in temporal patterns. This limitation should be discussed by the authors. 
5. How the method performs when one gene expression is a noisy realisation of the other one? Does the method fail to detect the similarity of these two expression trajectories? If yes, please discuss. More specifically, if  xbar(D)=xbar(C)+E and mean(E)=0; then xbar(D) is a noisy realisation of xbar(C) and the test should fail to reject the null hypothesis. However, by this method, we may get very large statistic and reject the null hypothesis. 
6.  In the second step, the proposed method treats time as quantitative variable and disregards the temporal order of measurements. According to the authors: “each gene’s expression profile over time was viewed as a gene-set”. What limitations will this feature impose to the analysis?

I have read this submission. I believe that I have an appropriate level of expertise to confirm that it is of an acceptable scientific standard, however I have significant reservations, as outlined above.

# Peer review: Referee response for version 1

**DOI:** 10.5256/f1000research.16734.r37528

**Published:** 2018-09-03

**Authors:** Shengping Yang

**Affiliations:** 1 Department of Pathology, Texas Tech University Health Sciences Center (TTUHSC), Lubbock, TX, USA

This study proposes to use a two-level SAMGSR method for feature selection in longitudinal microarray data analysis. The idea of considering the over-time expressions of a gene as the expressions of genes in a gene set is quite interesting. The following are a few concerns that can be further clarified in the revised version of this paper.

  

1. The longitudinal SAMGSR model, which has not been published before, was only briefly introduced. Since it has not been published, I would suggest to formally introduce the longitudinal model and include it as an alternative option in this paper.  
2. Data normalization is considered an important step in high throughput data analysis. It would great to provide a brief description.
3. The t-like statistic is used in SAMGSR, and the focus of this paper is microarray data. I am wondering if it is possible to have a very brief discussion on the feasibility of applying the proposed method to RNA-seq data after certain data transformation, e.g., “voom” normalization.
4. The inserted texts in Figure 1 do not seem to be consistent in size and font.
5. Please provide the link (and last access date) to the online Venn-diagram plot tool.
6. The first sentence in the abstract and introduction sections are identical. I would suggest making some minor modifications, e.g., using a more concise version in the abstract.

I have read this submission. I believe that I have an appropriate level of expertise to confirm that it is of an acceptable scientific standard.

# Peer review: Referee response for version 1

**DOI:** 10.5256/f1000research.16734.r36655

**Published:** 2018-08-17

**Authors:** Hulin Wu

**Affiliations:** 1 Department of Biostatistics and Data Science, School of Public Health, University of Texas Health Science Center at Houston (UTHealth), Houston, TX, USA

In this paper, authors intend to propose an extension of a pathway analysis method, SAMGSR, for longitudinal gene expression data. Both real data analysis and computer simulations are used to compare the modified method with the existing methods, which demonstrates some benefits of the proposed method. The proposed method is interesting, although the novelty is limited. Some other detailed comments and concerns are:

- More explanation on “a gene’s expression profiles over time can be considered as a gene set”.
- Right top on Page 2: When the SAMGSR is used for the first time, the reference should be provided.
- Bottom right on Page 2: The sample sizes (the number of patients) are inconsistent on two different paragraphs.
- English may need to be further polished.

I have read this submission. I believe that I have an appropriate level of expertise to confirm that it is of an acceptable scientific standard.
